# Validity and reliability of a Persian version of the self- evaluation of negative symptoms (SNS)

**DOI:** 10.1186/s12888-021-03521-7

**Published:** 2021-10-20

**Authors:** Shahrzad Mazhari, Anahita Karamooz, Mahin Eslami Shahrbabaki, Farzaneh Jahanbakhsh, Sonia Dollfus

**Affiliations:** 1 Neuroscience Research Center, Institute of Neuropharmacology, Kerman University of Medical Sciences, Kerman, Iran; 2 Department of Psychiatry, Shahid-Beheshti Hospital, Kerman University of Medical Sciences, P.O. Box: 76175- 113, Kerman, Iran; 3 Department of Psychiatry, Center Hospitalier Universitaire, 14000 Caen, France

**Keywords:** Negative symptoms, Persian, Psychometrics, Schizophrenia, SNS

## Abstract

**Aim:**

The Self-evaluation of Negative Symptoms (SNS) has been developed to allow schizophrenia patients to evaluate themselves in five dimensions of negative symptoms. The present study aimed to examine psychometric properties of the Persian version of SNS.

**Methods:**

A group of 50 patients with schizophrenia and a group of 50 healthy controls received the Persian-SNS. Severity of negative symptoms were evaluated by the Scale for Assessment of Negative symptoms (SANS) and the Brief Psychiatric Rating Scale (BPRS).

**Results:**

The results showed that the Cronbach’s alpha for the Persian SNS was 0.95. The Persian-SNS and its subscales showed significant positive correlations with the total SANS score and SANS subscales as well as BPRS negative subscale, thus confirming the validity of the scale. Finally, the Persian-SNS showed the ability to discriminate patients with schizophrenia from healthy controls.

**Conclusion:**

The acceptable properties of the Persian version of SNS demonstrated that it is a practical tool for screening negative symptoms in Persian-speaking schizophrenia patients.

## Introduction

Negative symptoms in patients with schizophrenia indicate the loss of certain behaviors or normal functioning, and are found at any stage of the disorder including: prodromal, acute, and residual phases. Negative symptoms usually persist even with improvements in positive symptoms, leading to poorer clinical outcome and quality of life [1].

Studies have reported severe negative symptoms in 26–36% of patients with schizophrenia [2]. Despite their high prevalence, recognizing negative symptoms is difficult due to secondary negative symptoms, which are caused by depression, antipsychotic adverse effects, social deprivation, or substance abuse [3, 4]. In recent studies, different standardized tools such as Brief Negative Symptom Scale (BNSS) and Clinical Assessment Interview for Negative Symptoms (CAINS) have been presented for a more effective treatment of negative symptoms [5, 6]. These instruments evaluate the severity of negative symptoms based on the evaluation of the observer, but not on the patient’s self-evaluation [7]. In fact, researchers believe that self-reporting complements objective evaluations of negative symptoms in schizophrenia, since patients are able to accurately express negative symptoms [7–10]. Moreover, patients’ self-evaluation is important because it allows them to evaluate their overall performance and participate in the process of examining and analyzing their symptoms, which in turn encourages them to participate more in the treatment process [11]. Finally, self-evaluation helps to identify negative symptoms at an early stage of the disorder [11].

Until now, a number of scales have been developed to assess the subjective experience of negative symptoms in patients with schizophrenia, including Subjective Experience of Deficits in Schizophrenia (SEDS) [12], Subjective Deficit Syndrome Scale [13], Subjective Experience of Negative Symptoms (SENS) [14], and Motivation and Pleasure Scale Self-Report (MAPSR). These scales have some limitations. For instance, the older scales are not self-assessment and the MAPRS fails to examine all the five dimensions [15].

Recently, Self-evaluation of Negative Symptoms (SNS) was developed by Dollfus et al. (2016) for schizophrenia patients and it has adequate psychometric properties of reliability and validity [11]. SNS has several advantages: it is short with 20 easily-understandable sentences and there are three options for each sentence which allows for a quick self-evaluation in only 5 minutes. Finally, SNS examines all the five dimensions of negative symptoms: motivation, asociality, alogia, anhedonia, and diminished emotional range [11].

To date, SNS has been translated into different languages including French [16], Lithuanian [17, 18], Spanish [19], Arabic [20] and Polish [21], and it has been validated with acceptable reliability and validity values. Persian, which is the official language of Iran, Afghanistan, and Tajikistan, is one of the most important languages of the Middle East and Central Asia. Over 110 million people speak Persian around the world and it is the 13th most spoken language in the world.

The aim of this study was to examine the reliability and validity of the Persian version of SNS in a group of Persian-speaking schizophrenia patients. It should be emphasized that all the methods were carried out in accordance with relevant guidelines and regulations.

## Methods

### Participants

A group of 50 Persian-speaking hospitalized schizophrenia patients (34 males) participated in the study. All the patients met the DSM-5 criteria for a lifetime diagnosis of schizophrenia based on diagnostic interview by two psychiatrists, and were receiving antipsychotic medications. The control group consisted of 50 healthy controls (34 males) with no personal or family history of psychotic illnesses. Exclusion criteria for both groups included history of head trauma, neurological disorders, and current substance dependency (within the past month). A written informed consent was obtained from all the participants as well as legally authorized representatives of human participants in the manuscript as Schizophrenia patients. The study was approved by the Ethics Committee of Kerman University of Medical Sciences (FR-622-28).

### Persian adaptation procedure

The standard forward-backward method was used for translation of SNS to Persian [22]. First, SNS was translated into Persian by two Persian-speaking psychiatrists. Second, these Persian versions were back translated into French by two independent bilingual experts who were blind to the original French version. In cases of incompatibility, an attempt was made to consider the underlying concepts rather than the literal translation. In the next step, the back-translated version was emailed to the original author (S. Dollfus). Finally, the back-translated version was amended and approved by Dr. Dollfus.

### Symptom assessment

SNS is self-evaluation of 20 items, evaluating the five areas of negative symptoms, giving each item a score of either 0 (strongly agree), 1 (somewhat agree), or 2 (strongly disagree). The total score ranges from 0 to 40, and the higher the score, the more severe the negative symptoms [16].

The Brief Psychiatric Rating Scale (BPRS) is a clinician-rated measure that assesses psychiatric symptoms. BPRS has 18 items which are rated on a seven-point Likert scale ranging from 1 (no sign) to 7 (severe). Higher scores indicate more severe psychopathology. Two sub scores can be determined for the positive (hallucinations, delusions, etc.) and negative (motor retardation, blunted affect, etc.) symptoms [18].

The Scale of Assessment of Negative Symptoms (SANS) has 25 items and measures the severity of negative symptoms in five subscales. Each item is rated from zero to five, and a higher score indicates a higher intensity of negative symptoms [19].

The Calgary Depression Scale for Schizophrenia (CDSS) is a nine-item semi-structured interview for assessing depressive symptoms in schizophrenia patients. Every item is rated on a four-point scale ranging from 0 (no symptom) to 3 (severe symptom) [23].

### Statistical analyses

Demographic and clinical data were compared between the two groups by using χ2-test for categorical variables and independent *t*-tests for continuous variables. Internal consistency was assessed by Cronbach’s alpha coefficient. Convergent validity was calculated using the Pearson correlation coefficient between the SNS total score and the BPRS negative score, and between the SNS subscores and the corresponding subscores of SANS. Sensitivity and specificity were calculated using receiver-operator curve (ROC), with all the patients coded as ‘cases’ and all the controls as ‘non-cases’.

## Results

Table 1 shows demographic and clinical characteristics of Persian study participants, who live in Iran. The two groups were well matched in terms of age, gender, and years of education. Schizophrenia patients had significantly higher SNS sub-scores as well as SANS and BPRS scores than the controls (Table 1).

**Table 1:** Demographic and clinical characteristics of Persian study participants, who live in Iran (mean ± SD)

		Patients *n* = 50	Controls *n* = 50	*p* value
Age (years)		39.5 ± 11.1	38.2 ± 11.2	0.90
Education (years)		9.8 ± 2.4	11.6 ± 2.2	0.07
Sex- N (% males)^a^		34 (68.0%)	33 (66.0%)	0.83
SNS	Social withdrawal	5.9 ± 2.7	0.86 ± 1.3	< 0.0001
	Decreased emotional range	5.7 ± 2.5	0.69 ± 1.1	< 0.0001
	Alogia	5.8 ± 2.5	0.53 ± 0.9	< 0.0001
	Avolition	6.1 ± 2.2	0.94 ± 1.2	< 0.0001
	Anhedonia	5.1 ± 2.5	0.41 ± 0.6	< 0.0001
	Total score	28.4 ± 11.0	3.4 ± 3.5	< 0.0001
SANS	Total score	71.1 ± 20.1	9.6 ± 8.9	< 0.0001
BPRS	Negative symptoms	8.1 ± 2.4	2.4 ± 0.8	< 0.0001
	Total score	40.1 ± 5.7	18.6 ± 3.0	< 0.0001
CDSS	Total score	6.2 ± 3.5	–	–
	CDSS score > 8 (%)	20	–	–
Length of illness (years)		9.4 ± 6.6	–	–
Age at onset (years)		19.3 ± 5.7	–	–
Mean chlorpromazine equivalent (mg)		468.7 ± 241.6		

*SNS* Self-evaluation of Negative symptoms, *SANS* Scale for the Assessment of Negative Symptoms, *BPRS* Brief Psychiatric Rating Scale, *CDSS* Calgary Depression Syndrome Scale, ^a^: χ^2^-test

### Reliability

Internal consistency for the 20 items of SNS was calculated using Cronbach’s alpha coefficient. The results showed excellent consistency with a_c_ = 0.95. Moreover, the inter-correlations between the five SNS sub-scores were significant (moderate to strong) in patients with schizophrenia (Table 2).

**Table 2:** Inter-correlations between the SNS sub-cores in schizophrenia patients

SNS sub-scores	Alogia	Avolition	Anhedonia	Social withdrawal	Diminished emotional range
Alogia	1	0.55**	0.67**	0.46**	0.76**
Avolition	0.55**	1	0.75**	0.58**	0.67**
Anhedonia	0.67**	0.75**	1	0.73**	0.73**
Social withdrawal	0.46**	0.58**	0.73**	1	0.64**
Diminished emotional range	0.75**	0.67**	0.73**	0.64**	1

** *p* < 0.01

### Validity

There were positive significant correlations between the SNS sub-scores and the SANS sub-scores, and between the SNS total score and BPRS negative sub-scores in schizophrenia patients (Table 3). However, there were not significant correlations between the SNS total score and the BPRS positive sub-score, or between the SNS total score and CDSS score (r = 0.09).

**Table 3:** Intercorrelations between the SNS sub-scores and SANS in schizophrenia patients

				SNS		
	Total score	Alogia	Avolition	Anhedonia	Social withdrawal	Diminished emotional range
BPRS-negative	0.70**					
SANS						
Total score	0.89**					
Alogia		0.78**				
Avolition			0.76**			
Anhedonia-Asociality				0.83**	0.70**	
Affect						0.71**

** *p* < 0.01, *SNS* Self-evaluation of Negative symptoms, *SANS* Scale for Assessment of Negative Symptoms, *BPRS* Brief Psychiatric Rating Scale

**Fig. 1:**
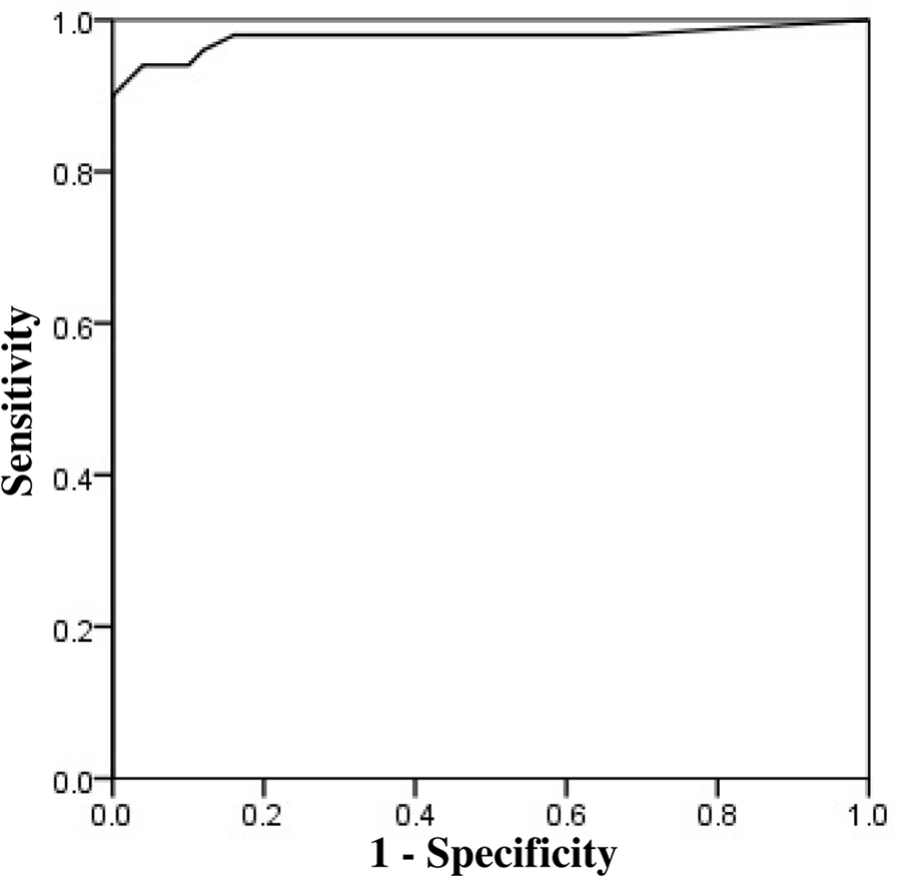
Receiver-operator curve for discrimination between schizophrenia patients and healthy controls

In this section, a ROC analysis was used to examine the contribution of the SNS total score to distinguish patients with schizophrenia from healthy controls. Herein, the area under ROC was 0.97 with 95% confidence interval (CI) = 0.94–1.0, *p* ≤ 0.001, sensitivity was 0.98, and specificity was 0.84 with the cut-off value of 7.5. As shown in Fig. 1, the results emphasize on the significant ability of the SNS total score for discriminating between schizophrenia patients and controls. Different thresholds according to sensitivity and specificity are shown in Table 4.

**Table 4:** Sensitivity and specificity of SNS for different threshold values

Threshold	Sensitivity	Specificity
4.5	0.98	0.64
5.5	0.98	0.68
6.5	0.98	0.80
7.5	0.98	0.84
8.5	0.96	0.88
9.5	0.94	0.90
10.5	0.94	0.96

## Discussion

Self-evaluation tools are important in order to empower schizophrenia patients to evaluate themselves in different dimensions of negative symptoms, and research and clinical works with an emphasis on negative symptoms of schizophrenia play an important role in this process. It seems necessary to introduce standard instruments with satisfactory psychometric features to countries and ethnicities with different languages. It should be mentioned that no self-evaluation tool for negative symptoms used in schizophrenia studies has been translated and validated into Persian language. In order to investigate the self-assessment of schizophrenia patients in the five negative domains of avolition, anhedonia, alogia, social withdrawal, and diminished emotional range, SNS can be considered as a valuable self-evaluation tool.

Overall, the results of this study showed that the Persian-SNS has high internal consistency, good convergent and discriminant validities, and a strong ability to distinguish patients with schizophrenia from healthy controls.

The results showed that the internal consistency of the Persian-SNS was good and similar to those of the French [16], Spanish [19], Arabic [20], and Polish [24] versions.

Similar to previous studies, there were significant correlations between the Persian- SNS subscales and the total score and subscales of SANS and BPRS-negative scale. The highest correlations were found between the total score of SNS with total score of SANS (r = 0.89) and asociality scales (r = 0.83). Similar results were found in the original paper (10) (Dolfus et al., 2016) and in another French sample [16]. This finding indicates the good convergent validity of the Persian-SNS and suggests that it is a useful tool for assessing negative symptoms in Persian-speaking schizophrenia patients.

In agreement with the Dolfus et al. study (2019) [25], the results of ROC analysis showed that the total score of Persian SNS differentiated patients with schizophrenia from controls, with sensitivity and specificity of 0.98 and 0.84, respectively. This result indicates that the Persian SNS is a valuable tool for discriminating between patients and controls based on negative symptoms.

There are some limitations to this study. First, test-retest reliability was not examined in this study, future research is required for assessing the reliability of the Persian-SNS over time. Second, relatively small number of patients participated in the study; however, this number is similar to that used by the original study for validation of the scale (49 patients).

In conclusion, the Persian SNS showed satisfactory reliability and validity, suggesting that it is a useful tool for evaluation of negative symptoms in clinical practice and research studies of the Persian-speaking people.

## Data Availability

The datasets used and/or analyzed during the current study are available from the corresponding author on reasonable request.
